# Differentiating external *zeitgeber* impact on peripheral circadian clock resetting

**DOI:** 10.1038/s41598-019-56323-z

**Published:** 2019-12-27

**Authors:** Isabel Heyde, Henrik Oster

**Affiliations:** Institute of Neurobiology, University of Lübeck, Lübeck, Germany

**Keywords:** Physiology, Feeding behaviour

## Abstract

Circadian clocks regulate physiological functions, including energy metabolism, along the 24-hour day cycle. The mammalian clock system is organized in a hierarchical manner with a coordinating pacemaker residing in the hypothalamic suprachiasmatic nucleus (SCN). The SCN clock is reset primarily by the external light-dark cycle while other *zeitgebers* such as the timing of food intake are potent synchronizers of many peripheral tissue clocks. Under conflicting *zeitgeber* conditions, e.g. during shift work, phase synchrony across the clock network is disrupted promoting the development of metabolic disorders. We established a *zeitgeber* desynchrony (ZD) paradigm to quantify the differential contributions of the two main *zeitgebers*, light and food, to the resetting of specific tissue clocks and the effect on metabolic homeostasis in mice. Under 28-hour light-dark and 24-hour feeding-fasting conditions SCN and peripheral clock, as well as activity and hormonal rhythms showed specific periodicities aligning in-between those of the two *zeitgebers*. During ZD, metabolic homeostasis was cyclic with mice gaining weight under synchronous and losing weight under conflicting *zeitgeber* conditions. In summary, our study establishes an experimental paradigm to compare *zeitgeber* input *in vivo* and study the physiological consequences of chronodisruption.

## Introduction

In most species, endogenous circadian clocks have evolved adjusting behaviour and physiology to anticipate daily recurring events, thus increasing the organism’s chance of survival. In mammals, ubiquitously expressed cellular clocks are organized in a hierarchical network^1^ coordinated by a central pacemaker residing in the hypothalamic suprachiasmatic nucleus (SCN)^2^. At the molecular level, circadian clocks are composed of interlocked transcriptional-translational feedback loops (TTLs) characterized by rhythmic transcription of clock genes including *brain and muscle aryl hydrocarbon receptor nuclear translocator-like protein 1* (*Bmal1* or *Arntl)*, *period* (*Per1-3)* and *D-site albumin promotor-binding protein (Dbp)*. The clock machinery controls cellular physiology through rhythmic activation of tissue-specific transcriptional programs.

External timing signals, so called *zeitgebers*, synchronise the internal clock network with external time. The most potent *zeitgeber* of the mammalian circadian system is light. The SCN integrates photic signals from the retina to reset subordinate clocks in other brain regions and peripheral tissues through innervation and humoral signals or through regulation of behavioural outputs such as the sleep/wake and feeding/fasting cycles^3–5^. Independent of the SCN, the timing of food intake can affect the circadian system. Restriction of food intake to the normal rest phase (*i*.*e*. the night in humans, the day in nocturnal rodents) uncouples peripheral tissue clocks from the SCN pacemaker and glucocorticoids play a role in this process^6,7^. Such desynchronisation of the internal clock network is a hallmark of shift work^8^ and may be a critical factor in the development of shift work-associated pathologies such as obesity, type-2 diabetes, cardiovascular, and mood disorders. To date, most animal studies investigate the effects of either abnormal T-cycles under *ad-libitum* feeding^9–11^ or time/calorie-restricted feeding regimes under normal 12 h-light:12 h-dark cycles^12–14^ on the circadian system. Furthermore, there are few studies combining restricted feeding with forced activity^15^. In humans, only non-invasive studies can be performed^16^. Dissecting the specific influence of different *zeitgebers* on tissue clock resetting, however, may help to design targeted interventions with the aim to adjust phase-synchrony within the circadian network, thus minimizing the risk of shift work-related diseases.

We here introduce a *zeitgeber* desynchrony (ZD) protocol in mice that allows quantifying the specific contributions of the two most potent circadian *zeitgebers*, light and food, to the resetting of different tissue clocks and the effect of *zeitgeber* uncoupling on metabolic homeostasis. Additionally, we conducted an inverse zeitgeber desynchrony experiment (iZD). To the best of our knowledge this is the first study investigating the impact of the two *zeitgeber* in this systematic way.

## Results

### Behavioural adaptation to conflicting *zeitgeber* conditions

**Figure 1:**
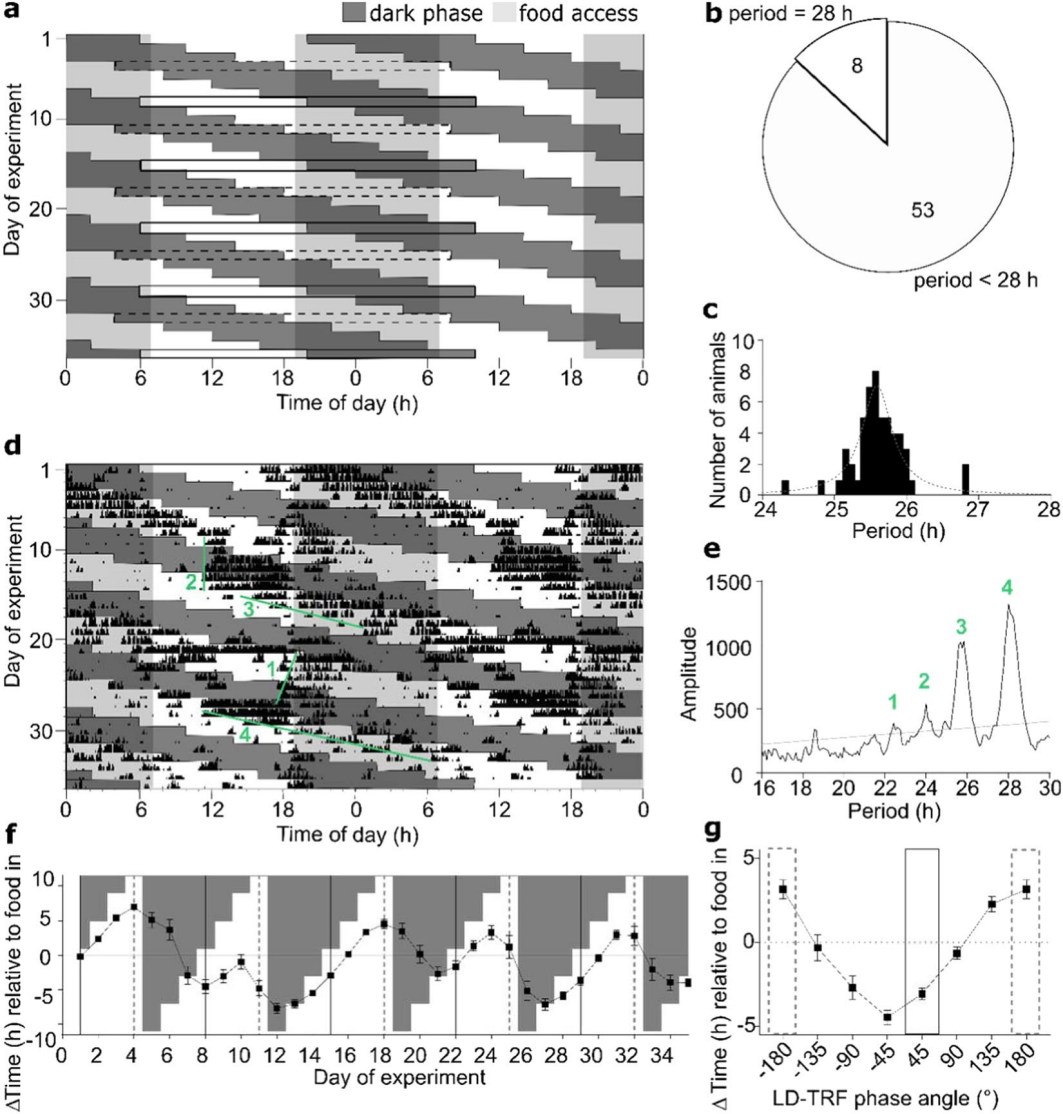
Locomotor activity under *zeitgeber* desynchrony (ZD) conditions. (**a**) Schematic representation of the ZD paradigm. Rectangles indicate in-phase (food access during the dark phase; solid frame) and anti-phase days (food access during the light phase; dashed frame). (**b**) Fractions of animals that completely adapted to the 28-hour LD cycle (period = 28 h; n = 8) and of free-running animals (period < 28 h; n = 53) under ZD conditions. (**c**) Distribution of dominant free-running periods (main periodogram peaks) under ZD conditions. The dotted curve shows a Lorentzian distribution fit (peak at 25.6 h). (**d**) Representative activity recording of one mouse over the course of 5 ZD cycles. Green lines depict different free-running rhythm components as indicated in (**e**). (**e**) χ^2^ periodogram analysis of running-wheel activity of the mouse shown in (**d**). Green numbers indicate free-run period components as depicted in (**d**). (**f**,**g**) Daily activity onset relative to feeding intervals throughout the experiment (**f**) and averaged activity onsets over one FD-cycle relative to the start of food access (**g**) during ZD. Three mice were excluded due to highly fragmented activity patterns. Data are shown as means ± SEM, n = 58. Dark phases are depicted by dark grey, food access by light grey shading.

To disentangle different *zeitgeber* inputs into the circadian system, mice were exposed to a *zeitgeber* desynchrony (ZD) paradigm combining a 28-hour light-dark (LD) cycle (14 h light: 14 h dark; LD-14) with a 24-hour time-restricted feeding paradigm (12 h food: 12 h no food; RF-12; Fig. 1a). Another cohort of mice were exposed to 24-hour light-dark (12 h light: 12 h dark, LD-12) combined with a 28-hour time-restricted feeding paradigm (14 h food: 14 h no food; RF-14; Fig. S6), defined as the inversed *zeitgebe*r desynchrony paradigm (iZD). During both protocols every 6^th^ experimental day animals experienced conditions when food access coincided with the dark phase. These days were defined as *in-phase* days since under *ad-libitum* conditions nocturnal mice also consume most their food during the dark phase^17^. Accordingly, days at which mice had food access almost exclusively in the light phase – thus being exposed to conflicting *zeitgeber* input – were defined as *anti-phase* days (Figs. 1a and S6). Mice exposed to the ZD protocol displayed a range of activity rhythms (Figs. 1b–e and S1). Roughly 13% of all mice (8 of 61) showed 28-hour activity rhythms phase-locked to the LD cycle (Figs. 1b and S1, top left). All other animals showed compound rhythms with median periods of 25.6 h falling between the 24- and 28-hour *zeitgeber* rhythms (Fig. 1c). χ^2^ periodogram analysis revealed up to four distinct period peaks (at 22–23 h, 24 h, 25–26 h, and 28 h) with a characteristic long period-short period sequence (Figs. 1d,e and S1). When determining the phase angle between activity onsets and the two *zeitgeber* rhythms, it became clear that activity regulation was a function of *zeitgeber* phase angle. The smallest phase difference between activity and feeding time was observed just prior to in-phase and largest on anti-phase days (Fig. 1f,g). These data suggest that behavioural rhythms are lengthened when phase differences between the *zeitgebers* become larger, but activity rhythms *snap back* (*i*.*e*. show short periods) when *zeitgeber* phases re-converge during the second part of the ZD cycle. In the iZD paradigm, locomotor activity adapted to the 12h-light:12h-dark cycles with very little activity in the light phase. Some animals showed increased activity in the light phase when it coincided with the end of the fasting phase (Figs. S6 and S7). Only two animals showed a dominant 28-hour adapted activity period under iZD conditions. Here, periodogram analysis revealed additional 24-hour activity periods for both animals (Fig. S6). Three animals of the 42 mice analysed showed additional free-running periods between the 24- and 28-hour *zeitgeber* rhythms in the iZD paradigm.

**Figure 2:**
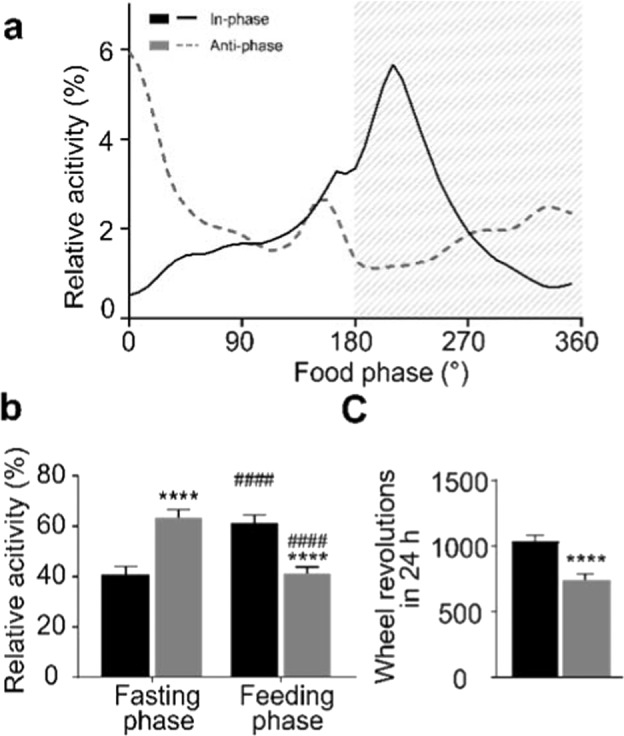
Activity profiles on ZD in-phase and anti-phase days. (**a**) Mean activity profiles at in-phase (solid black line) and anti-phase (dashed grey line) days relative to food access (180° = “food in”). (**b**) Feeding/fasting phase activity distribution at in-phase (black columns) and anti-phase days (grey columns; ****p < 0.0001 between days, ^####^p < 0.0001 between feeding and fasting phase on the same day, two-way ANOVA). (**c**) Total running-wheel activity at in-phase (black) and anti-phase days (grey; ****p < 0.0001, two-tailed, paired t-test). Values are means (±SEM in **b**,**c**), n = 60. One animal was excluded due to missing values in the activity recording of respective days.

Similar to standard LD conditions, running-wheel activity peaked in the beginning of the feeding/dark phase on in-phase days, though with a marked increase in activity towards the end of the light/fasting phase. On anti-phase days animals showed increased relative activity in the first half of the fasting/dark phase (Fig. 2a,b). The fasting/feeding phase distribution of activity patterns was almost inverted between in-phase (41 ± 3% fasting phase activity) and anti-phase days (63 ± 3% fasting phase activity, Fig. 2b). Overall, mice showed reduced total activity on anti-phase days (747 ± 41 *vs*. 1,031 ± 47 revolutions per day; Fig. 2c). Relative to the LD-14 cycle, running-wheel activity peaked in the beginning of the dark phase at 193° at both in-phase and anti-phase days (Fig. S2a,b).

### Shifting of clock gene expression rhythms in SCN and peripheral tissues under ZD conditions

**Figure 3:**
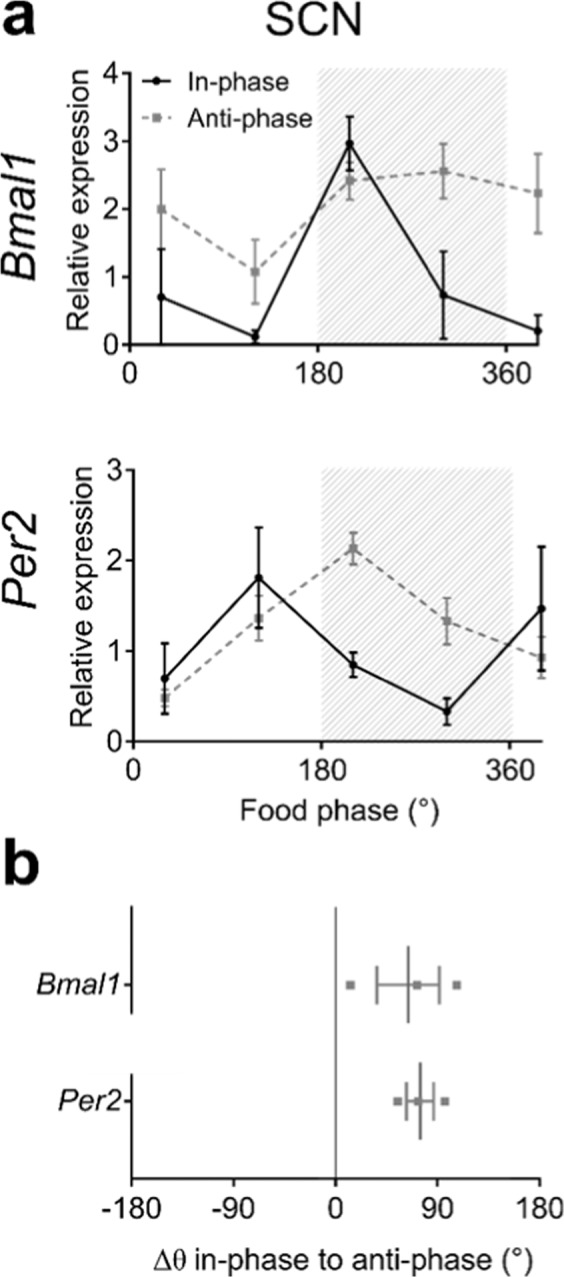
Regulation of SCN clock gene expression under ZD conditions. (**a**) Diurnal mRNA expression profiles for *Bmal1* (upper panel) and *Per2* (lower panel) on in-phase (solid black line) and anti-phase (dashed grey line) days determined by ^35^S-UTP *in situ* hybridisation. Data represent 3 independent experiments. Grey shading depicts time of food access (180° = “food in”). (**b**) Phase shifts of SCN clock gene expression rhythms between in-phase and anti-phase days. Data are shown as means ± SEM, n = 2–3 animals per time point.

In the SCN, mRNA expression profiles of two clock genes, *Bmal1* and *Per2*, were determined by ^35^S-UTP *in situ* hybridisation. In line with running-wheel activity rhythms, clock gene mRNA profiles in the SCN were phase-shifted relative to feeding time on anti-phase days compared to in-phase days (Fig. 3a,b). In addition, *Bmal1* expression patterns were dampened with overall high levels on anti-phase days (Fig. 3a, upper panel). Mean phase shifts between in-phase and anti-phase days were 64 ± 28° for *Bmal1* and 75 ± 12° for *Per2* (Fig. 3b).

**Figure 4:**
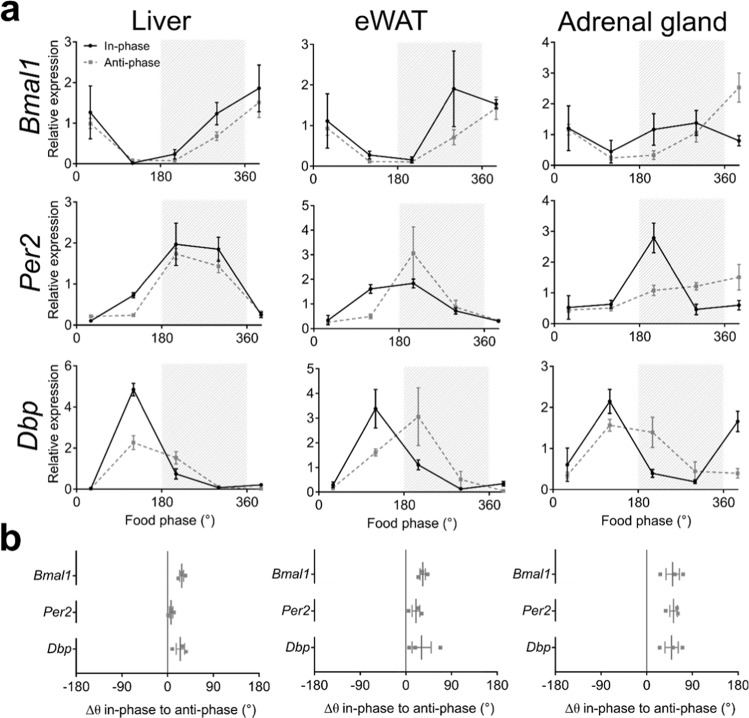
Regulation of clock gene expression in peripheral tissues under ZD conditions. (**a**) Diurnal mRNA expression profiles for *Bmal1* (top), *Per2* (middle), and *Dbp* (bottom) in three peripheral tissues on in-phase (solid black line) and anti-phase (dashed grey line) days determined by qPCR. Data represent 3 independent experiments. Grey shading depicts time of food access (180° = “food in”). (**b**) Phase shifts of peripheral tissue clock gene expression rhythms between in-phase and anti-phase days. Data are shown as means ± SEM, n = 2–4 animals per time point.

We next determined ZD clock gene (*Bmal1*, *Per2*, *Dbp*) mRNA expression profiles in three peripheral tissues (liver, epididymal white adipose tissue (eWAT), adrenal gland) by qPCR. Unlike the SCN, in peripheral tissues, clock gene expression profiles were similarly phased relative to feeding time – and, thus not to light phase – between in-phase and anti-phase conditions (Figs. 4a and S3–S4). In liver, phase differences were 28 ± 4°, 7 ± 3°, and 25 ± 8° for *Bmal1*, *Per2* and *Dbp*, respectively (Fig. 4b, left panel, Table S2). In eWAT, in-phase/anti-phase angles were slightly more pronounced at 33 ± 5°, 20 ± 8° and 31 ± 19° for *Bmal1*, *Per2* and *Dbp*, respectively (Fig. 4a,b, middle, Table S2). The largest clock gene expression phase shifts between in-phase and anti-phase days were seen in adrenals with 51 ± 13° (*Bmal1*), 53 ± 8° (*Per2*) and 49 ± 13° (*Dbp*) (Fig. 4a,b, right, Table S2). Variations between the different genes were small. Therefore, only one anti-phase profile was investigated under iZD conditions. Phase shifts were averaged over three genes for each tissue. Phase shifts were 10 ± 25° (liver), 55 ± 15° (eWAT) and 34 ± 12° (adrenal) between in-phase and anti-phase days (Fig. S8).

**Figure 5:**
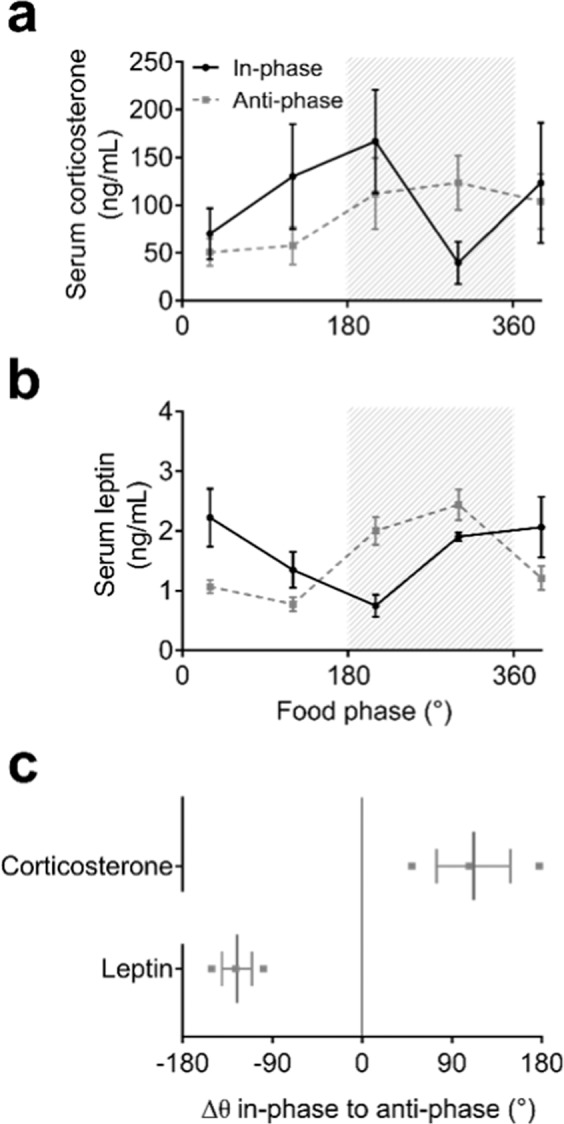
Corticosterone and leptin regulation under ZD conditions. (**a**) Diurnal serum corticosterone (**a**) and leptin (**b**) profiles on in-phase (black solid lines) and anti-phase days (grey dashed lines). Data represent 3 independent experiments. Grey shading depicts time of food access (180° = “food in”). (**c**) Phase shifts of corticosterone and leptin serum rhythms between in-phase and anti-phase conditions. Data are shown as means ± SEM, n = 2–4 animals per time point.

Finally, serum corticosterone and leptin level profiles were determined using radioimmunoassay (RIA) and enzyme-linked immunosorbent assay (ELISA), respectively (Figs. 5a,b and S5). The effect of ZD on the overall excretion for corticosterone and leptin was similar with mean phase shifts between in-phase and anti-phase days of 112 ± 37° and −125 ± 15°, respectively (Fig. 5c, Table S2).

### Regulation of energy metabolism under ZD conditions

**Figure 6:**
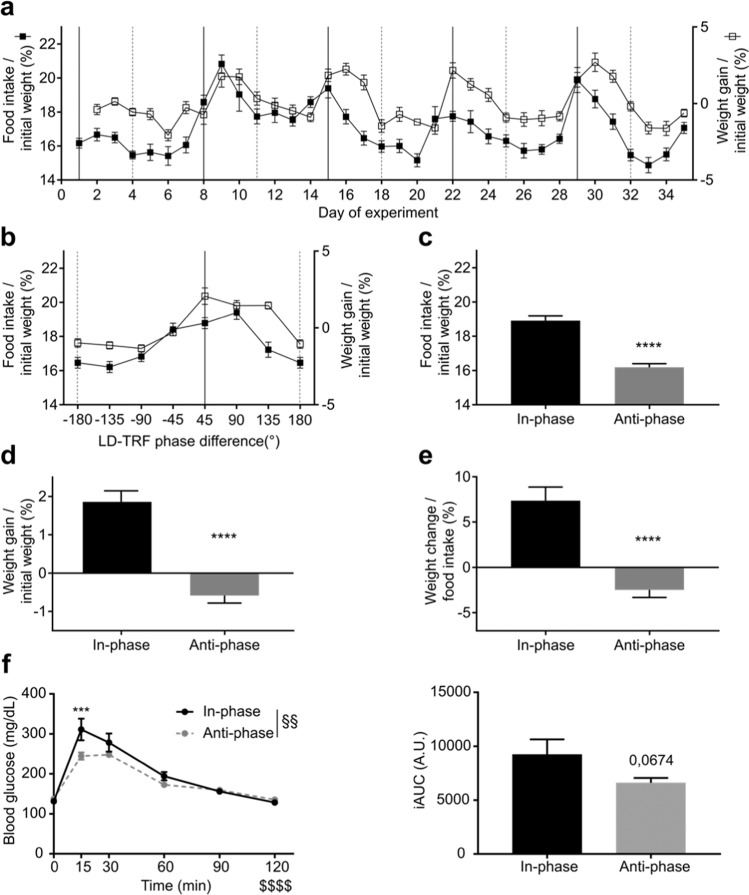
Changes in food intake and body weight under ZD conditions. (**a**) Food intake (closed squares) and body weight gain (open squares) throughout 5 weeks of the ZD paradigm. In-phase (solid black lines) and anti-phase days (dashed grey lines) are indicated. (**b**) Normalized food intake (closed squares) and weight change (open squares) profiles across the ZD cycle. (**c**) Normalized food consumption, (**d**) weight gain, and (**e**) energy conversion on in-phase and anti-phase days. All data are shown as means ± SEM (n = 45, ****p < 0.0001, two-tailed, paired t-tests). (**f**) Glucose tolerance test over time (left) and calculated area under the curve (right) on in-phase (n = 4) and anti-phase (n = 6) days. Due to technical problems two animals in the in-phase condition had to be excluded. Data are shown as means ± SEM, ***p < 0.001, condition effect ^§§^p < 0.005, time effect ^$$$$^ p < 0.0001, two-way ANOVA.

Our data so far revealed a tissue-specific adaptation of molecular clocks to ZD conditions with highest phase disruption on days of largest *zeitgeber* divergence. Such chronodisruption is also a hallmark of shift work and has been suggested to contribute to the increased risk of shift workers for metabolic disorders^18^. In line with this, under ZD conditions mice were also forced to consume food during the light phase (Fig. 1). To determine to which extent divergent *zeitgeber* input may affect metabolic homeostasis, we analysed food intake and body weight regulation under ZD conditions. Similar to what we had observed for running-wheel activity, animals showed cyclic changes in food intake and body weight regulation under ZD conditions (Fig. 6a,b). Food intake was highest around in-phase days whereas it declined and was lowest around anti-phase days. Mice consumed significantly more food on in-phase (18.8 ± 0.3% relative to body weight) than on anti-phase days (16.3 ± 0.2%; Fig. 6c). Body weight followed food intake. Animals lost weight on anti-phase days (−0.6 ± 0.2%) whereas they gained weight on in-phase days (1.9 ± 0.3%; Fig. 6d) resulting in markedly higher energy conversion rates (7.2 ± 1.5% *vs*. −3.7 ± 1.3%; Fig. 6e). Under iZD conditions animals showed cyclic changes in food intake and body weight regulation. Interestingly, the relations of food intake and body weight gain were inversed between in-phase and anti-phase days compared to the ZD paradigm. Under prolonged feeding-fasting cycles mice gained weight (0.8 ± 0.2% *vs*. −0.8 ± 0.3%) and consumed more food (23.4 ± 0.5% *vs*. 18.3 ± 0.6%) on anti-phase days compared to in-phase days, respectively. In contrast to the ZD-paradigm, food conversion was more effective on in-phase days under iZD conditions (−6 ± 1.5% *vs*. 3.1 ± 0.9%; Fig. S7b–e). Under ZD conditions blood glucose levels were higher at fifteen minutes after glucose injection on in-phase days but declined rapidly back to baseline levels (Fig. 6f, left). Animals showed improved glucose tolerance on anti-phase days under ZD conditions (Fig. 6f, right). Glucose tolerance was similar between in-phase and anti-phase days under iZD conditions (Fig. S7f). Body composition was comparable between the paradigms and between in- and anti-phase days of the same paradigm. Mice in the iZD-paradigm showed marginal increased fat mass (22.7% and 22.9% under iZD conditions *vs*. 20.9% and 20.5% under ZD conditions) and decreased lean mass (72.9% and 73.5% under iZD conditions *vs*. 75.6% and 74.2% under ZD conditions) compared to the animals of the ZD-paradigm, but the differences were not significant (Fig. S10).

### Central and peripheral tissue clock desynchrony under ZD conditions

**Figure 7:**
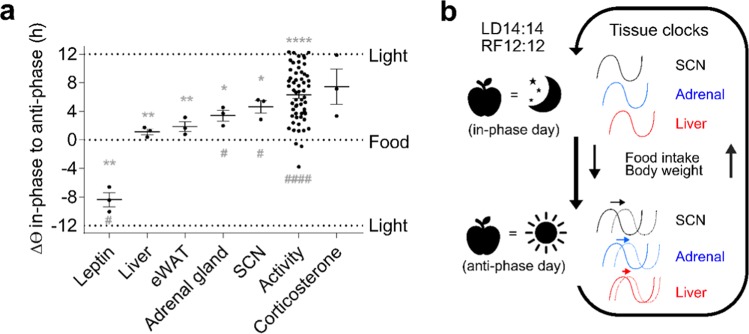
Determination of *zeitgeber* impact on different circadian rhythms under ZD conditions. (**a**) Phase shifts between ZD in-phase and anti-phase days for different endocrine, tissue clock, and behavioural rhythms conditions (means ± SEM, n = 3 for tissues/hormones, n = 58 for activity, one-sample t tests were used to determine statistical differences to the two *zeitgeber* rhythms (0 = feeding; ± 12 = light; ^*^p < 0.05, ^**^p < 0.005, ^****^p < 0.0001 *vs*. light, ^#^p < 0.05, ^####^p < 0.005 *vs*. food). (**b**) Model of circadian rhythm adaptation under conflicting *zeitgeber* (ZD) conditions with phase coherence on in-phase and maximal misalignment on anti-phase days.

In order to differentially quantify *zeitgeber* input into specific molecular and behavioural rhythms we compared in-phase/anti-phase day phase shifts across tissues/outputs (Table S1). For easier comparison, external time (equaling feeding time) was used as reference frame and phase shifts for each tissue were averaged across genes (Fig. 7a). From this perspective, a phase shift of 0 h would mean that the rhythm stays aligned with the feeding/fasting cycle (RF-12) while a phase shift of ±12 h would signify a rhythm phase-locked to the light-dark cycle (LD-14). With the exception of leptin showing a phase advance of *circa* 8 hours on anti-phase days, all rhythms studied aligned between the two *zeitgebers* with peripheral tissues aligning more closely with food (Fig. 7a). As would be expected, SCN clock gene expression and rest/activity cycles showed very comparable phase shifts which were significantly different from, both, light and food rhythms. On average, corticosterone rhythms were closest to the light-dark cycle. These data reveal a marked tissue/rhythm specificity of the two *zeitgebers* and allow a comparison of *zeitgeber* resetting capacity across different biological functions. Under inversed *zeitgeber* rhythms (LD-12,RF-14; iZD), peripheral tissue clocks show little phase shifts – but higher variance between the genes investigated – between in-phase days and anti-phase days (Figs. S8 and S9). Peripheral tissue clocks stayed aligned to the LD cycle under iZD conditions. Activity phase shifts were different from, both, light and food rhythms (Fig. S9). However, those phase shifts were much smaller under iZD compared to ZD conditions (Figs. 7a and S9). Thus, normal light-dark cycles are more potent to entrain behavioural rhythms and may stabilise peripheral tissue clocks at a 24-hour period when feeding-fasting cycles are out of the range of entrainment. In consequence, the strong impact of the entrained SCN on peripheral tissue clocks in the iZD paradigm does not allow the investigation of differential *zeitgeber* impact on various tissue clock resetting.

## Discussion

In the current study, we investigated the impact of diverging *zeitgebers* on the resetting of central and peripheral tissue clocks and humoral output. Under ZD conditions (a 28-hour LD cycle combined with a 24-hour feeding/fasting cycle), most mice showed intermediate activity patterns different from both *zeitgeber* periods. Most previous studies indicate that the range of entrainment to LD T-cycles varies between 21 h and 27 h in nocturnal rodents^19–21^. We chose a T-cycle which is out of the range of entrainment speculating that this would allow us to measure transient differences in period length regulation between different tissues. However, Usui and colleagues showed that rats adapted to a 28-hour LD cycle when period lengthening occurred stepwise^22^ and two recent studies suggest that mice can entrain to such periods even if T-cycle lengthening occurs immediately^21,23^. In our study, only 13% of mice adapted to LD-14. Unlike the other studies, in our ZD paradigm animals had no *ad-libitum* access to food. Therefore, conflicting inputs from the two *zeitgebers*, light and food, may have prevented full behavioural entrainment to either one external rhythm. Indeed, most mice displayed periods of activity dependent on the phase angle between the two *zeitgebers*. Such periodic variation of activity periods was also observed in other studies using short and long T-cycles in rodents with activity periods oscillating around that of the *zeitgeber*^9,24^. Together, these data suggest that both *zeitgebers*, light and food, impact on behavioural output at similar proportions, thus preventing complete adaption to one of them. In the iZD paradigm (a 24-hour LD combined with a 28-hour feeding/fasting cycle) only 5% of animals responded to the extended feeding-fasting cycles by entrainment at a 28-hour period. Those animals still showed some adaptation to the LD-12 cycle as periodogram analysis revealed a second activity period peak at 24 h. Thus, it appears likely that the LD-12 entrained the SCN pacemaker in all animals. Presumably, extended fasting periods lead to increased hunger and enhanced search for food resulting in slightly longer activity periods.

We compared phase regulation on days with in-phase (*i*.*e*. dark phase equals feeding phase) and anti-phase (*i*.*e*. light phase equals feeding phase) *zeitgeber* input to quantify the *pull* of each *zeitgeber* on molecular and behavioural rhythms. Analysis of the RF-12 timeframe yielded a reduction of total activity on anti-phase days due to reduced running-wheel activity during the feeding phase. On anti-phase days, mice displayed a small activity peak at the end of the fasting phase which could be interpreted as food anticipatory activity (FAA). However, this activity peak does not occur every day throughout the experiment as it would be expected for FAA. Furthermore, animals had access to food for 12 hours per 24 hours. FAA is usually only observable under restricted feeding with 4–8 h food access per day^25–27^. Under iZD conditions several mice exhibited increased activity at the end of the fasting phase especially when coming closer to the in-phase day. Here, mice were potentially seeking for food and were generally more aroused as their usual activity period is about to start.

In the SCN, phase shifts of clock gene expression rhythms were in the range of 5 h between in-phase and anti-phase days. Average phase shifts for *Bmal1* and *Per2* were very similar. In the SCN, *Per2* is directly light inducible leading to fast adaption of gene expression under jetlag conditions whereas *Bmal1* expression phase shifts slower^28–30^. In line with that, we observe little variation in *Per2* peak expression in the SCN. The observed higher variability in *Bmal1* peak expression might be a result of slow adaptation rates of *Bmal1* expression following light pulses^28^. Phase shifts between in-phase and anti-phase days in SCN clock gene expression and activity onset were similar underlining the importance of the SCN in driving behavioural outputs.

Peripheral tissues showed overall smaller phase shifts between in-phase and anti-phase days. Although the phase shifts were not significantly different when peripheral tissues were directly compared with each other, differential regulation relative to the two *zeitgeber* was observed. Liver and eWAT exhibited strongest correlation with the feeding cycle, whereas the adrenal gland showed a stronger influence by light in the ZD paradigm. Food access was previously shown to be a dominant *zeitgeber* for peripheral tissues^7,31^. Under iZD conditions activity – and thus presumably the SCN – stayed entrained to the LD cycle and peripheral tissue clocks were subsequently stabilised at a 24-h period, too. Regular light-dark cycles can affect peripheral tissue clocks without synchronisation by the SCN^5,32,33^. Our data support the idea that in mice, having a functional clock network, peripheral tissue clocks entrain to a *zeitgeber* rhythm which is in the range of entrainment in order to optimize metabolic yield. Under ZD conditions, phase shifts of clock gene rhythms in the adrenal were larger at around 4 h between in-phase and anti-phase days. Adrenal clocks are directly impacted by light^34^ and food^35^, which might explain the more balanced impact of both *zeitgebers* under ZD conditions. Phase shifts in the adrenal glands were indifferent to the LD cycle under iZD conditions supporting the idea of the stabilising effect by the entrained SCN.

Corticosterone levels rose at the end of the fasting phase on both days. Higher variations in the single sampling time points were seen on in-phase days and no difference in overall released corticosterone was found between in-phase and anti-phase days. Nevertheless, the amplitude of corticosterone appeared slightly dampened on anti-phase days. This suggest that the release of corticosterone from the adrenal might be impaired on anti-phase days when *Per2* gene expression in this tissue is dampened^36^. Glucocorticoids may themselves play a role in tissue clock entrainment in this context. Dexamethasone injections can reset the liver clock *in vivo*^37^ whereas no phase shifting effects were found in another study^31^. However, glucocorticoid signalling seems to be important for phase resetting in peripheral tissues^6,37,38^. It is reasonable to suggest that the dynamic changes we observe on the molecular and humoral level are a result of the integration of various inputs from central and peripheral tissue clocks^39^.

Leptin secretion is regulated by meal timing^40^ and by the SCN^41^. In our study, leptin levels were high in the early fasting phase and began to rise in the second half of the feeding phase on in-phase days. On anti-phase days leptin levels were low throughout the fasting phase and peaked in the first half of the feeding phase. These patterns suggest that mice ate late in the feeding phase on the day before the in-phase day which would induce a peak in leptin at the end of the feeding/beginning of the fasting phase. Leptin then dropped slowly to reach its minimum in the beginning of the feeding phase on in-phase days. On anti-phase days, leptin levels reached the nadir in the fasting phase suggesting that mice immediately started eating in the beginning of the feeding phase, consequently elevating leptin levels in the first half of the feeding phase. Conflicting inputs from the SCN may counteract regular feeding signals leading to large phase shifts through yet unknown mechanisms.

We investigated the metabolic state in both paradigms by analysing the daily changes in food intake and body weight. We further conducted glucose tolerance tests at the end of the fasting phase on in-phase and anti-phase days, respectively. Under ZD conditions mice showed cycles in metabolic homeostasis with weight gain on in-phase and weight loss on anti-phase days, respectively, with overall no weight gain throughout the experiment (data not shown). In contrast, under iZD conditions mice showed increased body weight on anti-phase days compared to in-phase days but also did not exhibit an overall weight gain throughout the experiment (data not shown). Similar oscillations were seen in the raw data (data not shown). However, the body weight reduction or increase was transient as mice started to consume more or less food when getting closer to the in-phase day in the ZD or iZD paradigm, respectively. Furthermore, body composition was similar between paradigms and in-between minimal and maximal *zeitgeber* phase alignment. Body weight composition may change in long-term ZD or iZD experiments. In both paradigms, mice were forced to eat in the light phase on anti-phase days. Daytime restricted feeding was shown to increase body weight under high-fat diet or chow^42,43^ but also prevented body weight increase compared to *ad-libitum* feeding^44^. Daytime food intake is unusual for nocturnal animals and might have caused a reduction of food intake under ZD conditions on anti-phase days and, consequently, weight. On the other hand, longer fasting periods may have induced overeating on anti-phase days under iZD conditions resulting in transient body weight gain especially around anti-phase days. Independent of the paradigm used we could not detect differences in total body weight gain throughout the experiment which may be due to changes in the daily food intake. Under ZD conditions glucose metabolism was impaired on in-phase days. This is surprising as other studies observe adverse effects on glucose metabolism under misaligned conditions^45^ and the mechanism would need further insight. It could be speculated that the dynamic *zeitgeber* phase relations in the ZD paradigm change the insulin sensitivity by differential impact on the pancreatic tissue clock. Furthermore, mice may spend more energy on anti-phase days and are in the need of glucose to replenish energy stores. The stabilisation of peripheral tissue clocks by the entrained SCN may prevent a misbalanced energy homeostasis and subsequent impairments in glucose metabolism.

Taken together, the entrainment of peripheral tissue clocks to restricted feeding does not drive food intake. Behavioural functions including food intake appear to be primarily driven by central clocks with only minor modulation by peripheral clocks. Our findings on food intake and body weight might be a result of dynamic changes in phase relations of feeding-fasting and dark-light and corresponding changes in the phasing of key enzymes, hormones and nutrients^46^.

Our study in a quantitative way compares the contribution of two major *zeitgebers* on central and peripheral tissue clock resetting. In the ZD paradigm, liver and eWAT clocks were primarily impacted by feeding-fasting (RF-12). Adrenal, SCN and activity were impacted by both *zeitgebers* showing phase shifts around 4 to 6 h between in-phase and anti-phase days. Corticosterone release was mostly impacted by light and may therefore contribute to shifts in behavioural outputs. A limitation of the ZD experiment is that some time points in the profiles of gene expression in the SCN and hormone levels were based on low replicate numbers (Table S1). Nevertheless, the data suggest a model of circadian rhythm adaption under dynamically conflicting *zeitgeber* conditions in which the circadian clock network de- and resynchronises along the ZD cycle. In line with this, food intake and body weight change throughout the ZD cycle reaching their troughs on days of maximal misalignment and peaking on in-phase days. The ZD paradigm allows for a much more fine-grained assessment of different *zeitgeber* inputs to circadian clocks and behaviour. In contrast, the iZD paradigm does not allow conclusions on differences in the *zeitgeber* impact on different tissue clocks. Presumably, under LD-12 conditions entrainment of the SCN and subsequent synchronisation signals are overwriting the *zeitgeber* signals from feeding-fasting cycles entraining behaviour and peripheral tissue clocks to the LD cycle. Therefore, future experiments may investigate the combination of shorter T-cycles for light-dark and lengthened feeding-fasting cycles to further disentangle differential *zeitgeber* impact on different tissues. It may, at a larger scale, allow for quantitively assessing chronodisruption and help devising tissue-specific manipulations of clock/rhythm function. Finally, our data suggest that time restricted feeding may promote synchrony between different peripheral tissues even under abnormal light cycles.

## Methods

### Animals

Young adult male C57BL/6J mice were maintained in the animal facility of the University of Lübeck. All experiments were in accordance with the German Law for Animal Protection and the FELASA guidelines for animal research. They were ethically assessed and legally approved by the MELUR Schleswig-Holstein, Germany. *Prior* to experiments mice were kept under 12-hour light: 12-hour darkness conditions (LD-12) with 300 lux illumination during the light phase and access to chow food (Altromin #1314) and water *ad libitum*.

### *Zeitgeber* desynchrony (ZD) paradigms

Mice were singly housed in running-wheel cages. Animal age ranged from 11–17 weeks at the start of the ZD paradigm and one experiment lasted between 4–8 weeks. After 1 week of acclimatisation, mice were put under 14-hour light: 14-hour darkness (LD-14) conditions with 300 lux illumination at light phases while food access was given at 12-hour food access: 12-hour fasting (RF-12) intervals (Fig. 1a). On the first day of the experiment food was removed 1 h after “lights on” and accessible again 1 h before “lights off”. Each day mice and food were weighed *prior* to food access.

In an inverse experiment (iZD), another cohort of mice (n = 48) were kept under 12-hour light:12-hour darkness (LD-12) conditions with 300 lux illumination at light phases while food access was provided at 14-hour food access:14-hour fasting (RF-14) intervals. On the first day, food was removed from the hopper 1 h before “lights on” and accessible again 1 hour after “lights off”. Each day food and mice were weighed *prior* to food access.

### Behavioural measurements

Running-wheel usage of singly-housed mice was recorded and analysed using the ClockLab system and software (6.0.34, Actimetrics, Evanston, USA). Activity recordings started during acclimatisation to ensure that mice were adapted to the light-dark cycle and to the running-wheels. Mice were accustomed to handling for several days before the start of the experiment. During the experiment mice were weighed before the access to food and consumed food was measured.

### Tissue and serum collections

On in-phase or anti-phase days animals were sacrificed at specified times (*zeitgeber* times (ZT) 2, 8, 14, and 20, and 2 again (not included in analysis) relative to food access; “food in” = ZT12; n = 2–5 per time point). Note that under iZD conditions animals were sacrificed at comparable time points along the fasting-feeding cycle (e.g. “ZT2” = 2.3 h after food out). Mice were sacrificed by cervical dislocation followed by immediate decapitation. In the dark phase sacrificing was performed under dim red light and eyes were removed before turning on the lights for tissue dissection. Trunk blood was collected and kept at room temperature for 30 min for clotting and then centrifuged for 30 min at 664 × g and 4 °C. Serum was stored at −20 °C. Whole brains were isolated and fixed in 4% paraformaldehyde (PFA) in phosphate buffered saline (PBS) for 12–18 h before dehydration in ascending ethanol series and subsequent embedding in paraffin. Liver, eWAT and adrenals were stored in RNA*later* (ThermoFisher, Waltham, USA) at −20 °C before RNA isolation.

### RNA isolation and quantitative real-time (q)PCR

Tissues were homogenised using a bench homogenizer (Biolab, Bebensee, Germany) and total RNA was extracted with TRIzol reagent (ThermoFisher). cDNA was obtained from isolated RNA using random-hexamer primers and High-capacity cDNA Reverse Transcription Kit (Applied Biosystems, Foster City, USA) following the manufacturer’s protocol. cDNAs were diluted 1:10–1:20 and stored at −20 °C. qPCR was done using Go-Taq qPCR Master Mix (Promega, Madison, USA) on a Bio-Rad CFX96 thermocycler (Bio-Rad, Hercules, USA). The following primers were used: *Eef1a* forward 5′-TGCCCCAGGACACAGAGACTTCA-3′; *EeF1a* reverse 5′-AATTCACCAACACCAGCAGCAA-3′; *Bmal1* forward 5′CCTAATTCTCAGGGCAGCAGAT-3′; *Bmal1* reverse 5′-TCCAGTCTTGGCATCAATGAGT-3′; *Per2* forward 5′- GCCAAGTTTGTGGAGATTCCTG-3′; *Per2* reverse 5′-CTTGCACCTTGACCAGGTAGG-3′; *Dbp* forward 5′-AATGACCTTTGAACCTTGATCCCGCT-3′; *Dbp* reverse 5′-GCTCCAGTACTTCTCATCCTTCTGT-3′.

### Radioactive *in situ* hybridisation

Paraffin embedded brains were cut at 8 µm on a microtome (ThermoFisher). *In situ* hybridisation with ^35^S-UTP labelled antisense RNA probes was performed as described^47^. S^35^-UTP was purchased from PE (Perkin Elmer, Waltham, USA). *Bmal1* and *Per2* riboprobes were synthesized with T7 and T3 RNA polymerases, respectively^48,49^. Hybridisation was performed overnight at 50 °C. Quantification was determined by densitometric analysis of autoradiography films (Amersham Hyperfilm MP, GE Healthcare, Chicago, USA). Films were developed using an automatic developing machine (Kodak, Rochester, USA). Developed films were scanned in a high-resolution scanner (Bio-Rad) and grey-scale images were quantified using the Quantity One software (Bio-Rad) for expression levels. Regions of interest were normalized to background.

### Immunoassays

Serum leptin was measured by ELISA (#EZML-82K, Millipore, Burlington, USA) following the manufacturer’s protocol. Serum corticosterone levels were determined using ImmuChem Double Antibody Coticosterone ^125^I RIA Kit (#07120102, MP Biomedicals, Eschwege, Germany) according to the manufacturer’s protocol.

### Glucose tolerance test (GTT)

GTTs were conducted in the third cycle of ZD or iZD on in-phase or anti-phase days. Animals were only used once. Mice were fasted for 12 h prior to GTT. A total of six animals was used for each GTT. Blood glucose was measured before the start of GTT using a glucometer (ACCU-CHECK Aviva, Roche, Basel, Switzerland). Intraperitoneal injection of 1.5 g glucose in 0.9% NaCl/kg body weight was was followed by blood glucose determination at 15, 30, 60, 90 and 120 minutes after injection.

### Body composition

Body composition was analysed using nuclear magnetic resonance (minispec LF110, Bruker, Billerica, USA) after the end of glucose tolerance test in the fasted mice. Free body fluid, fat and lean mass were measured. Fat and lean mass were normalized to individuals’ body weight (n = 6 for each condition).

### Data analyses and statistics

χ^2^ periodogramm analysis of activity recordings (n = 61 (ZD) or 42 (iZD)) was conducted excluding the first 3 experimental days. Peaks above p < 0.05 were considered as statistically significant. 30-min bin size running-wheel rotations were extracted for analysis using ClockLab version 6.0.34 (Actimetrics). Total running-wheel activity was averaged over 4–6 in-phase and anti-phase days, respectively, for 28 h (relative to light) or 24 h (relative to food). Activity was individually normalized to total activity for each in-phase and anti-phase day. Normalized activity of 4–6 in-phase and anti-phase days were averaged and displayed as relative activity, respectively. Feeding/fasting and light/dark phase distributions were calculated from relative activity profiles. In the relative activity profiles time is presented in degrees for LD and RF whereas 0° is “lights on”/“food out” and 180° is “lights off”/“food in”, respectively. Activity profiles were smoothed using a 3-neighbour running-average in GraphPad Prism 7 (GraphPad, San Diego, USA). Light and dark activity sections of anti-phase days were inverted for easier comparison of phase angles.

Daily activity onsets were determined by three independent investigators on long-term, double plotted actograms. Investigators were unaware of the phase relation of the ZD cycle. In case of disagreements, two additional investigators were asked to determine activity onset. The activity onset times of at least two investigators had to match for usability in further analysis.

Daily food intake was normalized to the fasted body weight of the animal. Body weight was normalized to the body weight of the previous day. Food conversion rate was determined by division of the body weight change by food intake of the same experimental day.

Gene expression was normalized to *eukaryotic elongation factor-1 α* (*Eef1a*). Relative expression ratios (using the ΔΔCT method) were calculated^50^ and normalized to the mean ratio of the corresponding in-phase profile. Likewise, hormone levels were normalized to the mean ratio of in-phase profiles. Overall hormone excretion and individual gene expression between in-phase and anti-phase conditions were tested for significant differences using unpaired, two-tailed t tests. Three independent anti-phase profiles (ZD paradigm) or one anti-phase profile (iZD) were used for subsequent phase shift analysis using the centre of gravity (COG) as reference. The mean expression curves were analysed using CircWave 1.4^51^ and COGs were determined. Phase shifts of analysed genes (3 for peripheral tissues and 2 for the SCN) of each anti-phase profile were averaged to estimate tissue clock overall phase shifts. Differences of hormonal COGs between in-phase and anti-phase profiles were calculated. For activity phase shifts 3–5 in-phase to anti-phase transitions of individual activity onsets were averaged.

Two-way ANOVA was used to test for significant differences in activity distribution. Differences in activity peak of in-phase to anti-phase conditions relative to light or food phase were analysed using two-tailed, paired t tests, respectively. Food intake differences and body weight changes between in-phase and anti-phase days were analysed using two-tailed, paired t tests. For GTT analysis, two-way ANOVA was used to test for significant differences between conditions and Sidak’s multiple comparisons tests were performed to evaluate differences between conditions at specific time points. Areas under the curve for GTTs were analysed using two-tailed, paired t-tests. Statistical differences of body composition were analysed by one-way ANOVA (testing between paradigms) or two-tailed, unpaired t tests (between in-phase and anti-phase day of the same paradigm). Statistical differences of phase shifts between in-phase and anti-phase conditions were analysed in one-sample t tests against hypothetical values of minimal (0 h) and maximal (12 h) phase shifts. Rhythmicity of diurnal profiles was assessed using CircWave (s.a.). p-values below 0.05 were considered significant.

## Supplementary information

Dataset 1

## Data Availability

The datasets generated and analysed during the current study are available from the corresponding author on reasonable request.
