# Supplementary material for: Differentiating external zeitgeber impact on peripheral circadian clock resetting
Source: Sci Rep. 2019 Dec 27;9:20114. doi: 10.1038/s41598-019-56323-z (PMC6934673; doi:10.1038/s41598-019-56323-z)
Supplement: Supplementary file 1 — Dataset 1 [file 41598_2019_56323_MOESM1_ESM.docx]

**Title:** Differentiating external *zeitgeber* impact on peripheral circadian clock resetting

**Authors:** Isabel Heyde & Henrik Oster

**Supplementary data**


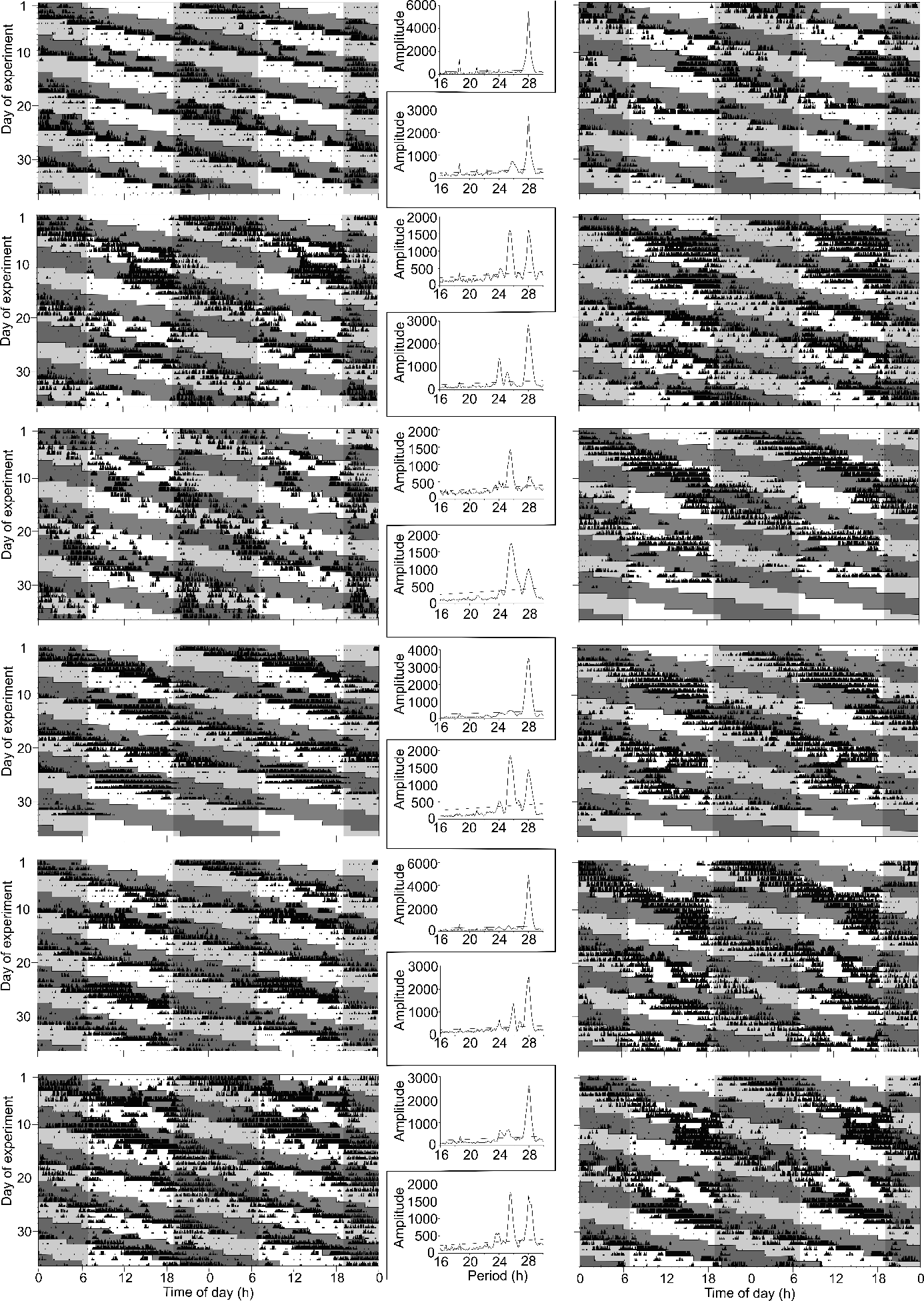
**Figure S1**. Activity recordings and periodogram analyses of mice under ZD conditions. Representative double plotted long-term actograms and corresponding ꭓ^2^ periodogram analyses are shown. Dark grey shades represent dark phases, light grey shades represent feeding interval.


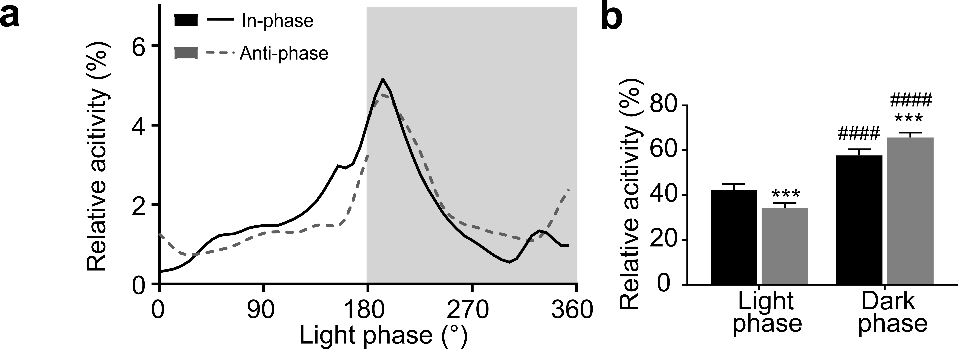


**Figure S2.** Activity profiles on ZD in-phase and anti-phase days. a) Mean activity profiles at in-phase (solid black line) and anti-phase (dashed grey line) days relative to light (180 ° = “lights off”). b) Light/ dark phase activity distribution at in-phase (black) compared to anti-phase days (grey; *** p = 0.0002 between days, #### p<0.0001 between feeding and fasting phase on the same day, two-way ANOVA). Values are means (± SEM in b), n=60.

**Table S1.** Sample sizes per time point used for COG determination for gene expression in SCN and hormone level in serum. Sample sizes below three are indicated in red.

| SCN | | | | | | | | | | |
| --- | --- | --- | --- | --- | --- | --- | --- | --- | --- | --- |
| Bmal1 | | | | |  | Per2 | | | | |
| ZT (Food) | In-phase | Anti-phase 1 | Anti-phase 2 | Anti-phase 3 |  | ZT (Food) | In-phase | Anti-phase 1 | Anti-phase 2 | Anti-phase 3 |
| 2 | 3 | 2 | 3 | 2 |  | 2 | 3 | 3 | 3 | 3 |
| 8 | 3 | 3 | 3 | 2 |  | 8 | 3 | 3 | 3 | 3 |
| 14 | 3 | 3 | 3 | 2 |  | 14 | 3 | 3 | 3 | 2 |
| 20 | 3 | 3 | 2 | 3 |  | 20 | 3 | 3 | 3 | 3 |
|  |  |  |  |  |  |  |  |  |  |  |
|  |  |  |  |  |  |  |  |  |  |  |
| Hormones | | | | | | | | | | |
| Leptin | | | | |  | Corticosterone | | | | |
| ZT (Food) | In-phase | Anti-phase 1 | Anti-phase 2 | Anti-phase 3 |  | ZT (Food) | In-phase | Anti-phase 1 | Anti-phase 2 | Anti-phase 3 |
| 2 | 3 | 2 | 3 | 3 |  | 2 | 3 | 3 | 3 | 3 |
| 8 | 3 | 3 | 3 | 3 |  | 8 | 3 | 3 | 3 | 3 |
| 14 | 4 | 3 | 2 | 3 |  | 14 | 4 | 3 | 2 | 3 |
| 20 | 2 | 3 | 3 | 2 |  | 20 | 4 | 3 | 3 | 2 |

**Table S2.** COGs of gene expression and hormone levels in peripheral and central tissue and serum, respectively. COGs are presented as decimal hours (external time).

|  |  | In-phase | Anti-phase 1 | Anti-phase 2 | Anti-phase 3 |
| --- | --- | --- | --- | --- | --- |
| Liver | Bmal1 | 22.02 | 0.35 | 23.80 | 23.45 |
|  | Per2 | 16.30 | 16.79 | 16.37 | 15.46 |
|  | Dbp | 8.77 | 11.18 | 10.76 | 9.38 |
| eWAT | Bmal1 | 21.45 | 0.30 | 23.64 | 23.07 |
|  | Per2 | 12.85 | 14.91 | 14.43 | 13.20 |
|  | Dbp | 9.34 | 13.87 | 10.57 | 9.77 |
| Adrenal | Bmal1 | 20.16 | 0.93 | 23.86 | 21.93 |
|  | Per2 | 13.71 | 17.83 | 17.70 | 16.22 |
|  | Dbp | 7.59 | 12.30 | 11.03 | 9.27 |
| SCN | Bmal1 | 15.01 | 15.88 | 19.80 | 22.17 |
|  | Per2 | 8.39 | 13.24 | 14.83 | 12.04 |
| Corticosterone |  | 12.21 | 19.36 | 0.07 | 15.55 |
| Leptin |  | 2.24 | 17.80 | 19.64 | 16.20 |


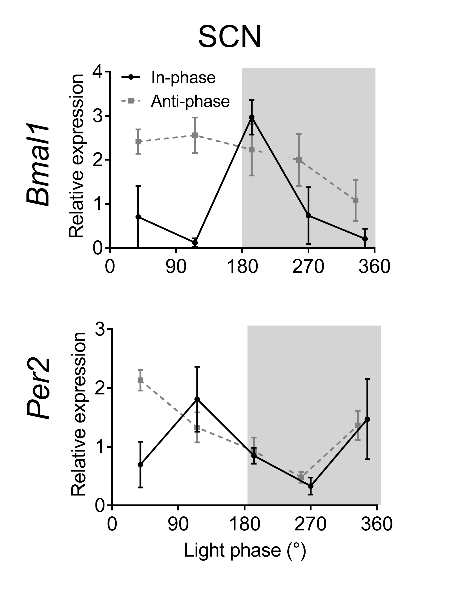


**Figure S3.** Regulation of SCN clock gene expression under ZD conditions. a) Diurnal mRNA expression profiles for *Bmal1* (upper panel) and *Per2* (lower panel) on in-phase (solid black lines) and anti-phase (dashed grey lines) days relative to light determined by ^35^S-UTP *in situ* hybridisation. Anti-phase profiles represent the average of 3 independent experiments. Grey shading depicts lighting conditions (180 ° = “lights out”). Data are shown as means ± SEM, n=2-3 animals per time point.


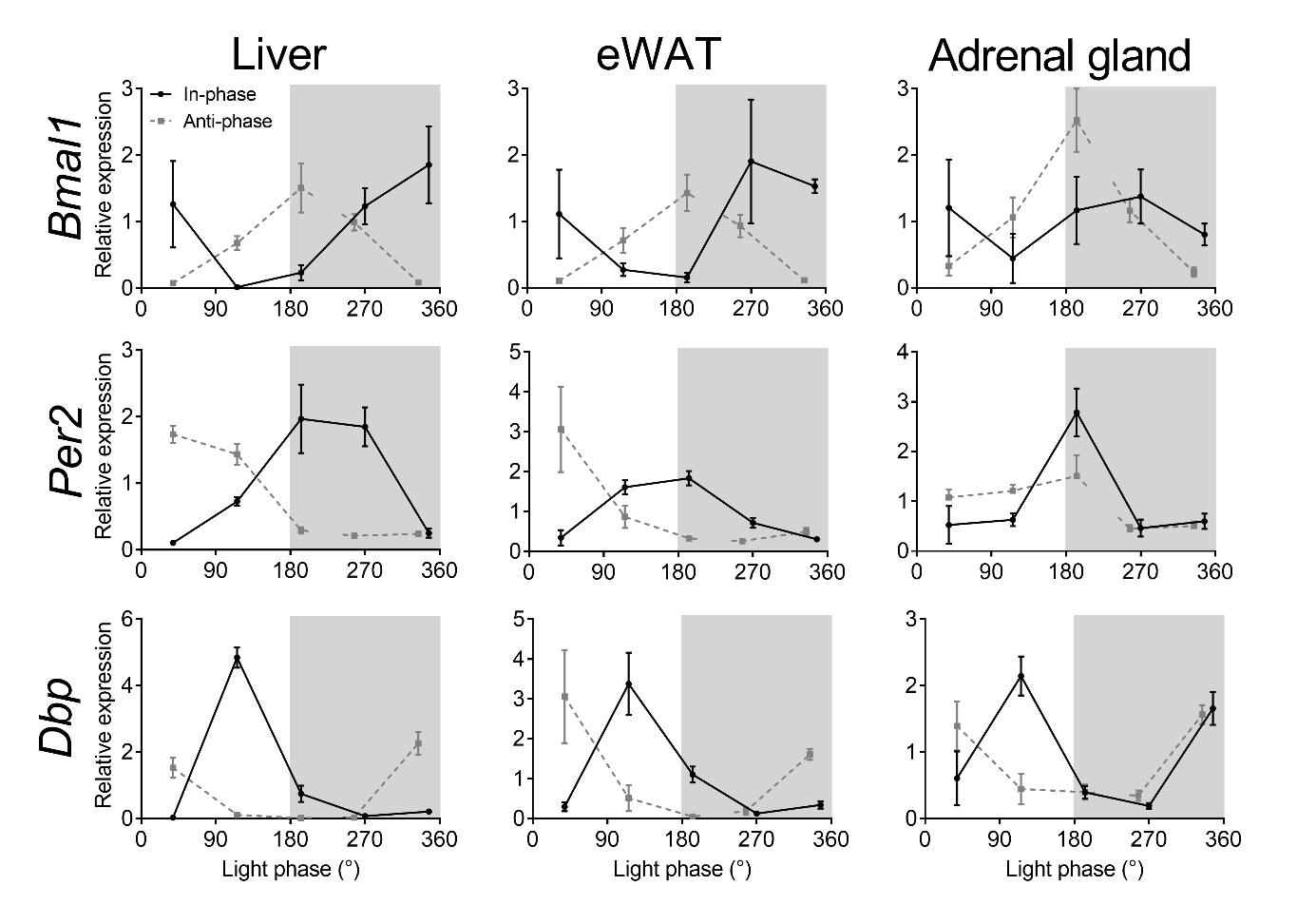


**Figure S4.** Regulation of clock gene expression in peripheral tissues under ZD conditions. a) Diurnal mRNA expression profiles for *Bmal1* (top), *Per2* (middle), and *Dbp* (bottom) in three peripheral tissues on in-phase (solid black line) and anti-phase (dashed grey line) days determined by qPCR relative to light. Anti-phase profiles represent the average of 3 independent experiments. Grey shading depicts lighting conditions (180 ° = “lights out”). Data are shown as means ± SEM, n=2-4 animals per time point.


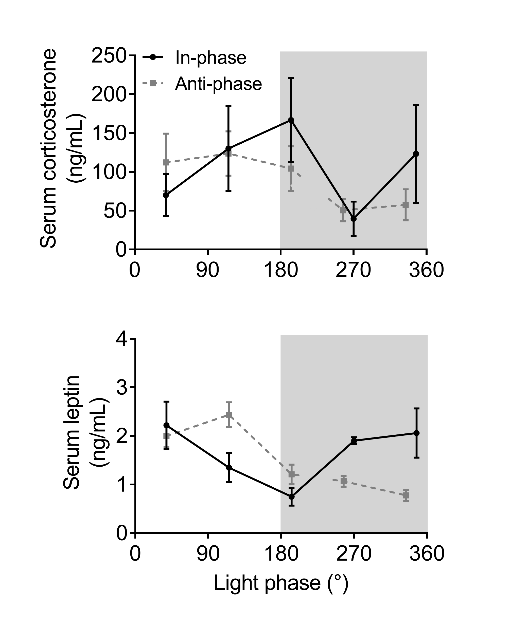


**Figure S5.** Corticosterone and leptin regulation under ZD conditions. a) Diurnal serum corticosterone (upper panel) and leptin (lower panel) profiles on in-phase and anti-phase days relative to light. Data represent 3 independent experiments. Grey shading depicts lighting conditions (180 ° = “lights off”). Data are shown as means ± SEM, n = 2-4 animals per time point.


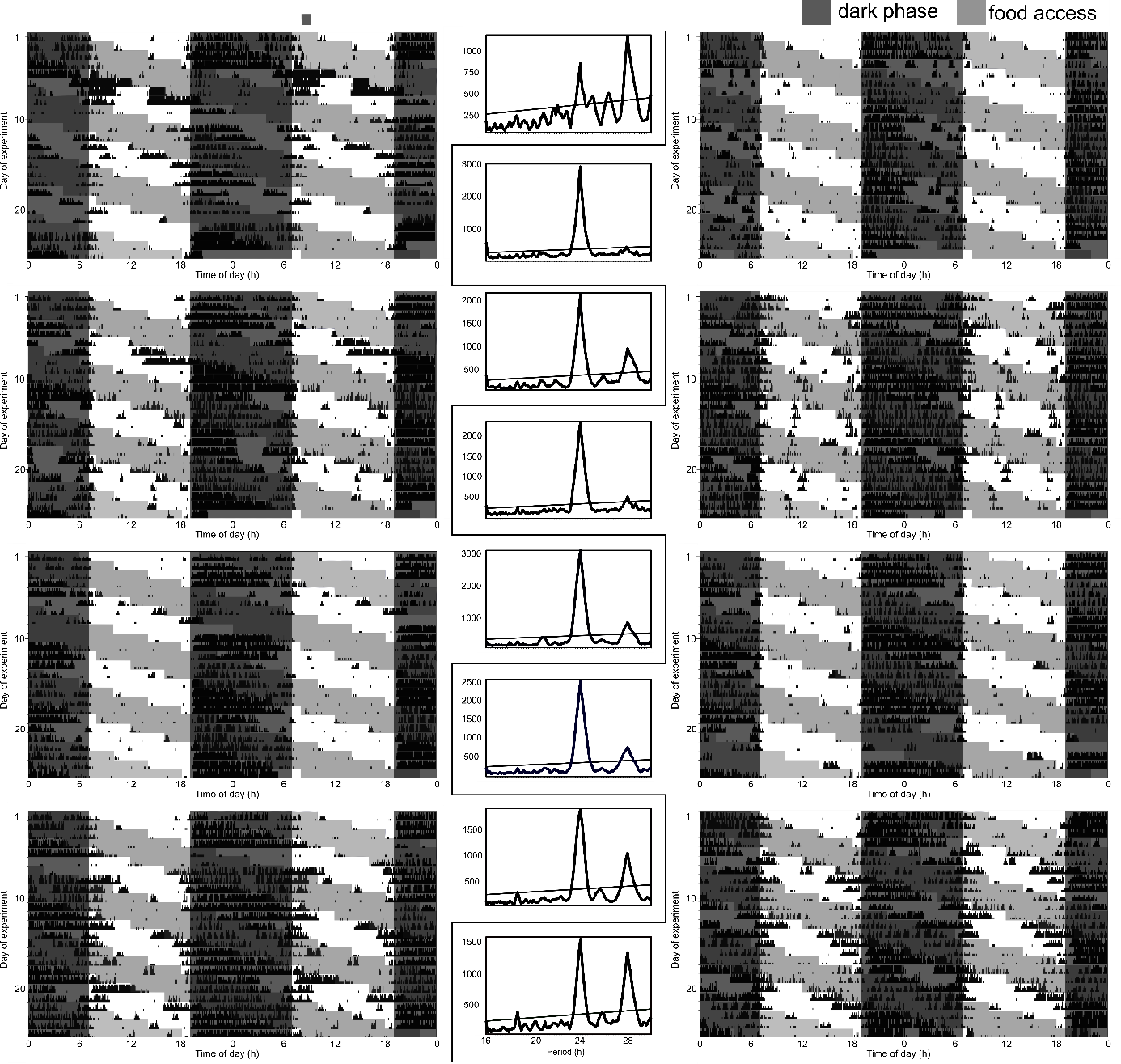


**Figure S6**. Activity recordings and periodogram analyses of mice under iZD conditions. Representative double plotted long-term actograms and corresponding ꭓ^2^ periodogram analyses are shown. Dark grey shades represent dark phases, light grey shades represent feeding interval.

**
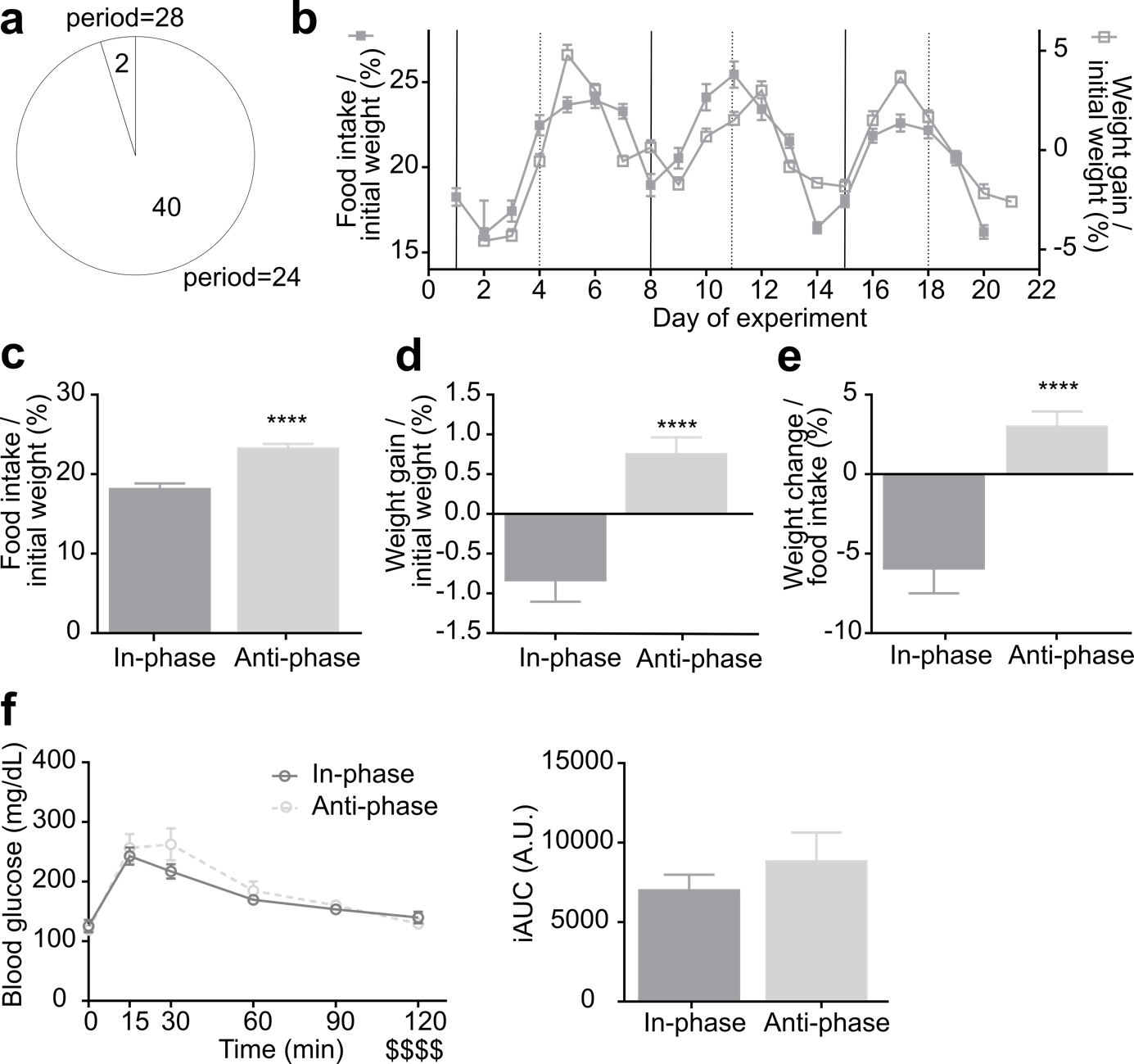
**

**Figure S7.** Activity periods and changes in food intake and body weight under iZD conditions. a) Fractions of animals showing activity periods of 24 h or 28 h in Χ^2^-periodogramm analysis b) Food intake (closed squares) and body weight gain (open squares) throughout 3 weeks of the iZD paradigm. In-phase (solid black lines) and anti-phase days (dashed grey lines) are indicated. c) Normalized food intake (closed squares) and weight change (open squares) profiles across the iZD cycle. d) Normalized food consumption, e) weight gain, and f) energy conversion on in-phase and anti-phase days. All data are shown as means ± SEM (n=48, **** p<0.0001, two-tailed, paired t-tests). f) Glucose tolerance test over time (left) and calculated area under the curve (right) on in-phase (n=6) and anti-phase (n=5) days under iZD conditions. One animal of the in-phase day had to be excluded due to a technical problem. Data are shown as means± SEM, time effect $$$$ p<0.0001, two-way ANOVA.


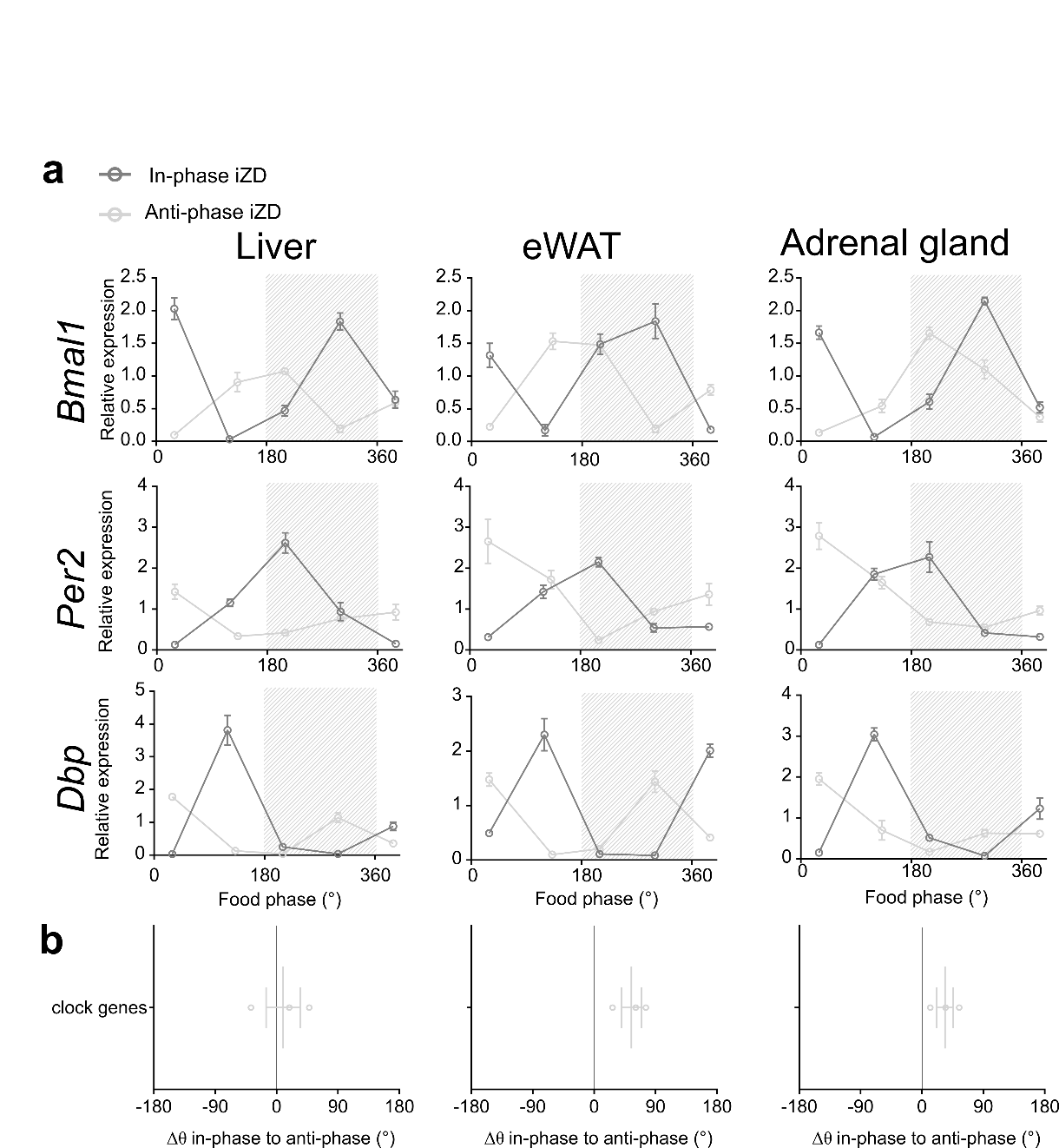


**Figure S8.** Regulation of clock gene expression in peripheral tissues under iZD conditions. a) Diurnal mRNA expression profiles for *Bmal1* (top), *Per2* (middle), and *Dbp* (bottom) in three peripheral tissues on in-phase (dark grey line) and anti-phase (light grey line) days determined by qPCR. Grey shading depicts time of food access (180 ° = “food in”). 360 ° correspond to 28 h. b) Phase shifts of peripheral tissue clock gene expression rhythms between in-phase and anti-phase days. Data are shown as means ± SEM, n=3-5 animals per time point.

**Table S3.** COGs of gene expression in peripheral tissue. COGs are presented as decimal hours (external time).

|  |  | In-phase | Anti-phase |
| --- | --- | --- | --- |
| Liver | Bmal1 | 7.48 | 8.93 |
|  | Per2 | 23.023 | 20.11 |
|  | Dbp | 16.5 | 20.23 |
| eWAT | Bmal1 | 7.06 | 12.97 |
|  | Per2 | 20.63 | 22.73 |
|  | Dbp | 16.73 | 21.49 |
| Adrenal | Bmal1 | 5.59 | 8.29 |
|  | Per2 | 21.51 | 22.46 |
|  | Dbp | 15.55 | 19.81 |


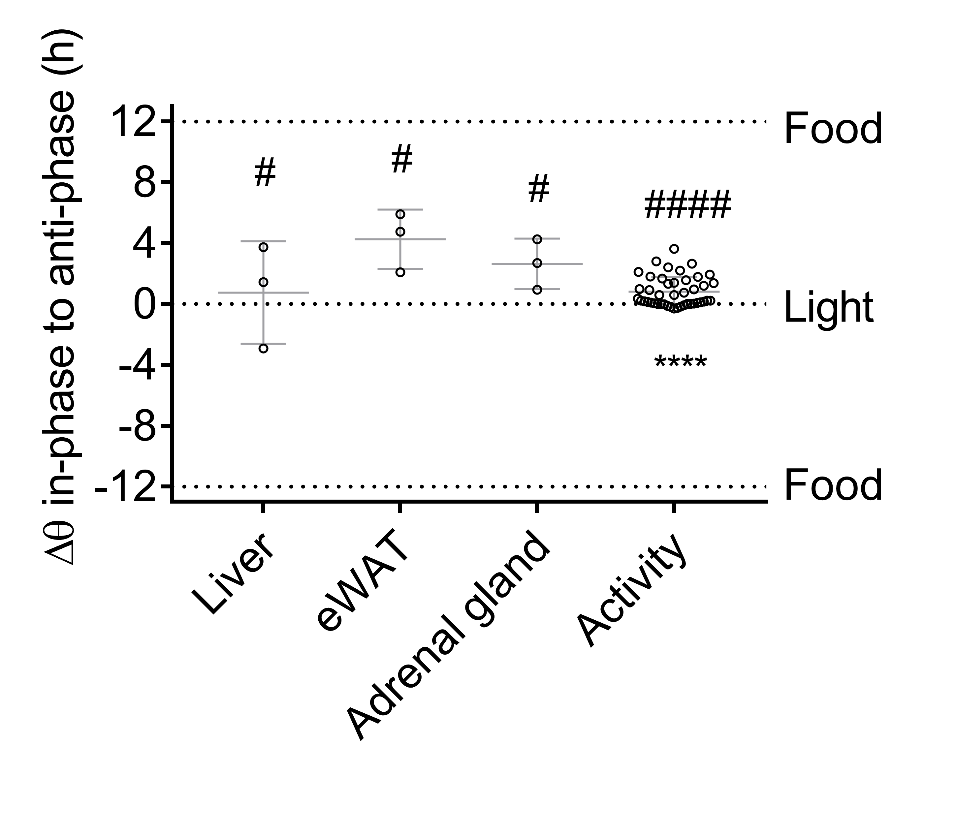


**Figure S9.** Determination of *zeitgeber* impact on different circadian rhythms under iZD conditions. a) Phase shifts between iZD in-phase and anti-phase conditions for different tissue clocks and behavioural rhythms (means ± SEM, n = 3 for tissues, n = 42 for activity, one-sample t-tests were used to determine statistical differences to the two *zeitgeber* rhythms (0 = light; ± 12 = food; # p<0.05, #### p<0.0001 *vs.* food, **** p<0.0001 *vs.* light).


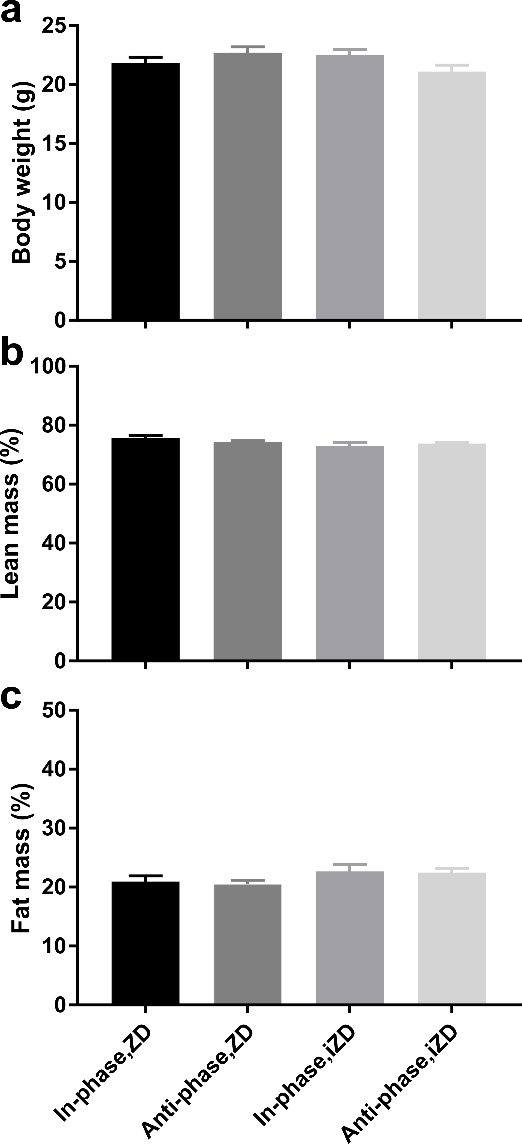


**Figure S10.** Body composition on in-phase and anti-phase days under ZD and iZD conditions. a) Body weight, b) normalized lean mass, and c) normalized fat mass (means ± SEM, n = 6 for each condition). One animal in the anti-phase iZD condition has to be excluded due to technical reason.
